# Delta-like 1-mediated cis-inhibition of Jagged1/2 signalling inhibits differentiation of human epidermal cells in culture

**DOI:** 10.1038/s41598-019-47232-2

**Published:** 2019-07-25

**Authors:** Victor A. Negri, Meike E. W. Logtenberg, Lisa M. Renz, Bénédicte Oules, Gernot Walko, Fiona M. Watt

**Affiliations:** 1 Centre for Stem Cells and Regenerative Medicine, Faculty of Life Sciences & Medicine, King’s College London, 28th Floor, Tower Wing, Guy’s Hospital, SE1 9RT London, UK; 2 Present Address: Division of Immunology, The Netherlands Cancer Institute, Postbus 90203, 1006 BE Amsterdam, The Netherlands; 3 Present Address: Research Institute for Applied Bioanalytics and Drug Development, IMC University of Applied Sciences, A-3500 Krems an der Donau, Austria; 4 Present Address: Department of Biology and Biochemistry, University of Bath, Claverton Down, Bath, BA2 7AY United Kingdom

**Keywords:** Cell signalling, Skin stem cells

## Abstract

Epidermal homeostasis depends on a balance between self-renewal of stem cells and terminal differentiation of their progeny. Notch signalling is known to play a role in epidermal  stem cell patterning and differentiation. However, the molecular mechanisms are incompletely understood. Here we demonstrate dynamic patterns of Notch ligand and receptor expression in cultured human epidermis. Notch2 and 3 act together to promote differentiation, while Notch1 decreases stem cell proliferation. The Notch ligand Jagged1 triggers differentiation when presented on an adhesive substrate or on polystyrene beads and over-rides the differentiation inhibitory effect of cell spreading. In contrast, Delta-like 1 (Dll1) overexpression abrogates the pro-differentiation effect of Jagged1 in a cell autonomous fashion. We conclude that Dll1 expression by stem cells not only stimulates differentiation of neighbouring cells in trans, but also inhibits differentiation cell autonomously. These results highlight the distinct roles of different Notch receptors and ligands in controlling epidermal homeostasis.

## Introduction

Mammalian epidermis comprises a multi-layered epithelium, termed inter-follicular epidermis (IFE), with various associated appendages including hair follicles, sebaceous glands, and sweat glands^1^. Maintenance of the IFE and its appendages depends on several distinct stem cell (SC) populations^1–4^. IFE SCs reside in the basal cell layer of the epithelium where they are anchored to an underlying basement membrane^1^. They divide to produce SCs that remain in the basal cell layer or cells that are destined to undergo terminal differentiation in the suprabasal cell layers^1,4,5^. Once terminal differentiation has been triggered, committed progenitors (CPs) detach from the basement membrane and become sorted into the suprabasal layers. There, they undergo a programmed series of morphological and biochemical changes that include the synthesis of the differentiation-specific keratins K1 and K10, as well as involucrin (IVL), transglutaminase (TGM) 1, periplakin (PPL), and loricrin^6^.

The Notch signalling pathway plays an important role in regulating epidermal homeostasis^7,8^. Notch receptors are initially synthesized as single precursor proteins, which are subsequently cleaved during transport to the cell membrane by a Furin-like convertase at site S1^8^. S1 cleavage generates two subunits (extracellular domain (ECD) and transmembrane and intracellular domain (TMICD)) which are held together noncovalently at the cell surface^8^. Notch signalling is activated via interaction with ligands (such as Jagged1, Jagged2 and Dll1) that are themselves transmembrane proteins and results in two successive proteolytic cleavages of the Notch receptor^7,8^. The first cleavage is mediated by an ADAM family metalloprotease (tumor necrosis factor-α-converting enzyme, TACE), which cleaves the receptor at site S2, close to the transmembrane domain of the TMICD, to generate the Notch extracellular truncation (NEXT) fragment^7,8^. The second cleavage is mediated by γ-secretase and occurs intracellularly within the transmembrane domain of the NEXT fragment at site S3, resulting in the release of the Notch intracellular domain (NICD) into the cytoplasm^7,8^. The NICD translocates into the nucleus and forms a complex with a transcriptional coactivator, leading to the activation of Notch target gene transcription^7,8^.

Genetic ablation or activation of the Notch pathway has revealed that a key function of epidermal Notch signalling is promotion and maintenance of the differentiated cell state^7,8^. However, there is ambiguity about the precise roles of the different Notch receptors, as both specific and redundant signalling functions have been reported^9–12^. Likewise, it is unknown whether, as in other tissues, different Notch ligands transduce distinct signals by preferentially engaging with specific receptors^13–15^. All Notch ligands likely have dual functions, as exemplified by Dll1: it initiates Notch signalling in a neighbouring cell in-trans and initiates a PDZ-dependent signalling mechanism in-cis^16^. The complexity of Notch signalling is further increased by the phenomenon of cis-inhibition, a mechanism whereby Notch ligands engage with receptors in the same cell to inhibit or dampen Notch signals transduced in-trans via Notch ligands on the surface of neighbouring cells^17,18^. A higher tendency of Dll1 null keratinocytes to initiate terminal differentiation^19^ could indicate that such a mechanism is operating in the IFE SC compartment.

In this study, we demonstrate that different Notch receptors and ligands play overlapping but distinct roles in controlling terminal differentiation in the human IFE and provide new mechanistic insights into how Notch signalling is regulated to maintain a balance between SC renewal and differentiation.

## Results

### Dynamic expression of Notch signaling components in cultured human epidermis

**Figure 1:**
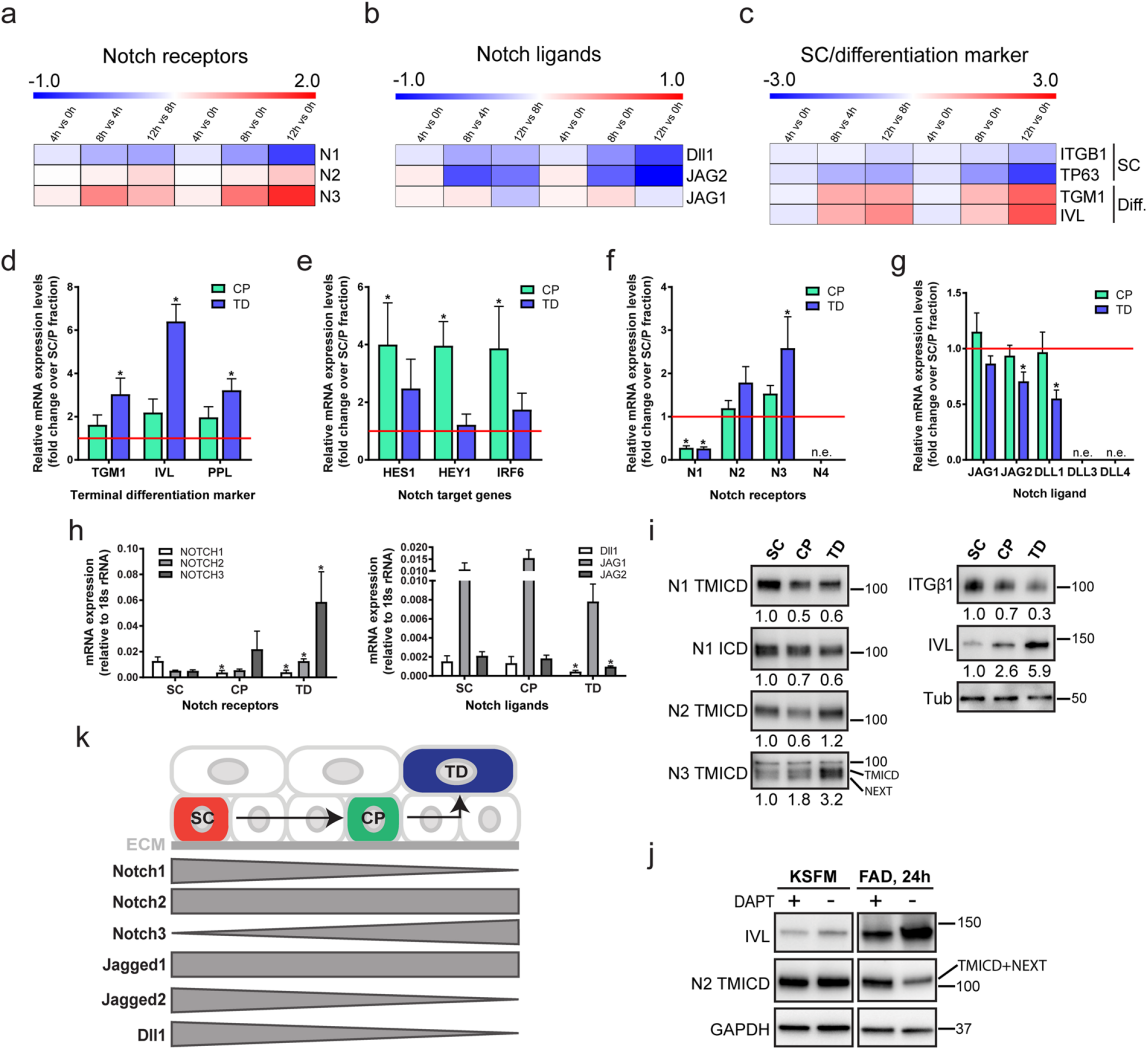
Dynamic expression pattern of Notch signalling components in human keratinocyte cultures. (**a–c**) Heatmaps showing Log_2_ fold change of normalised gene expression for pairwaise comparisons of mRNA levels of Notch receptors (Notch1(N1), Notch2(N2), Notch3(N3)) (**a**), Notch ligands (Delta-like1(Dll1), Jagged1(JAG1), Jagged2(JAG2) (**b**), and stem cell (SC)/terminal differentiation markers (**c**) during suspension culture. For each condition the mean of n = 3 independent replicates in the microarray dataset was used and the pairwise fold change comparison was between the means of both samples. (**d–h)** Q-RT PCR analysis of mRNA levels of terminal differentiation markers (**d**), Notch signalling target genes (**e**) and Notch receptors and ligands (**f–h**) in enriched populations of stem- (SC), committed progenitor- (CP), and terminally differentiated (TD) cells. Data shown are from n = 5 independent experiments. Bars in (**d–g**) represent the average fold change in mRNA abundance (normalised to 18sRNA) compared to the SC-enriched fraction (red line) in each experiment. Bars in (**h**) represent mean ΔCq expression. Error bars represent S.D. P-values were calculated using one-way ANOVA with Holm Sidak’s multiple comparisons test (*p < 0.05). (**i**) Western blots of enriched keratinocyte populations (as in **d–h**) using antibodies against the TMICD and NEXT fragments of Notch1, 2 or 3, the ICD of Notch1, integrin (ITG)β1, or involucrin (IVL). Tubulin was used as loading control. Numbers below lanes represent normalised protein ratios relative to an arbitrary level of 1.0 set for the SC-enriched fraction. Note that the Notch1 and Notch2 TMICDs and NEXT fragments cannot be separated sufficiently by SDS-PAGE to appear as discreet bands. (**j**) Western blots of keratinocytes cultured in KSFM or FAD, in the presence or absence of DAPT, using antibodies against the TMICD and NEXT fragment of Notch2, or IVL. GAPDH was used as loading control. Note accumulation of the Notch2 TMICD and NEXT fragment upon DAPT treatment in differentiating cells, indicating Notch2 receptor activation upon differentiation commitment. (**k**) Schematic illustration summarising expression patterns of different Notch receptors and ligands. ECM: extracellular matrix.

When human epidermal SCs are deprived of extracellular matrix (ECM) adhesion by culturing them in suspension (0 h), they rapidly transit through a commitment stage (4 h) to undergo terminal differentiation by 12 h in suspension^20^. By examining a previously published microarray dataset^20^, we found that Notch1 levels declined in suspension, while there was a moderate upregulation of Notch2 from 8 h to 12 h and strong induction of Notch3 from 4 to 12 h. (Fig. 1a). Expression of the terminal differentiation markers IVL and TGM1 increased from 8 h, whereas SC markers (ITGB1, TP63)^5,20^ were downregulated (Fig. 1c). Expression of the Notch ligands Dll1 and Jagged2 decreased in suspension, whereas Jagged1 remained relatively constant during the first 8 h of suspension-induced differentiation (Fig. 1b). Similar changes in Notch1/2/3, Dll1 and Jagged1/2 expression are observed during terminal differentiation of developing mouse interfollicular epidermis^21^.

To assess whether the observed changes in Notch receptor and ligand expression also occurred in adherent cultures, we disaggregated preconfluent stratified colonies of human keratinocytes and enriched for SC (rapidly adherent), CP (slowly adherent) or terminally differentiating (non-adherent) cells by differential adhesion to ECM^22^ (Fig. 1d). Q-RT PCR analysis of the Notch signalling target genes HES1, HEY1 and IRF6^23^ showed that, relative to SC, expression was highest in the CP-enriched fraction (Fig. 1e). Notch1 was the predominant receptor expressed by epidermal SCs (Fig. 1f,h), while expression of Notch3 was upregulated in terminally differentiated cells (Fig. 1f,h), consistent with the induction of expression in suspension (Fig. 1a). Jagged1 was the major Notch ligand expressed in all cell fractions, and Dll1 and Jagged2 levels declined during terminal differentiation (Fig. 1g,h). Notch4, Dll3 and Dll4 were not detected (Fig. 1f,g).

We also examined the relative levels of total and activated Notch proteins in the different cell fractions by western blotting with antibodies to the TMICD or NICD (generated by S3 cleavage) of Notch1, the TMICD of Notch2, or the TMICD and NEXT fragment (generated by S2 cleavage) of Notch3^24^ (Fig. 1i). Antibody specificity was confirmed by probing lysates of cells expressing shRNAs for each receptor (Suppl. Fig. 1a,b). The level of activated Notch1 declined in CP and differentiated cells relative to SC, whereas the levels of Notch3 increased, consistent with the changes in mRNA levels. Notch2 levels were lower in CP than in the other populations, but this was not reflected in decreased mRNA levels (Fig. 1f,h,i), potentially indicating strong receptor activation leading to a decrease in the levels of the TMICD^24^. Indeed, inhibition of S3 cleavage using the γ-secretase inhibitor DAPT led to accumulation of the Notch2 TMICD and NEXT fragment in cells grown in standard culture medium (FAD), but not in low calcium medium (KSFM), and also resulted in decreased IVL expression (Fig. 1j). The dynamics of Notch receptor and ligand expression are summarised schematically in Fig. 1k.

### Distinct and and overlapping effects of Notch receptors and ligands on differentiation

**Figure 2:**
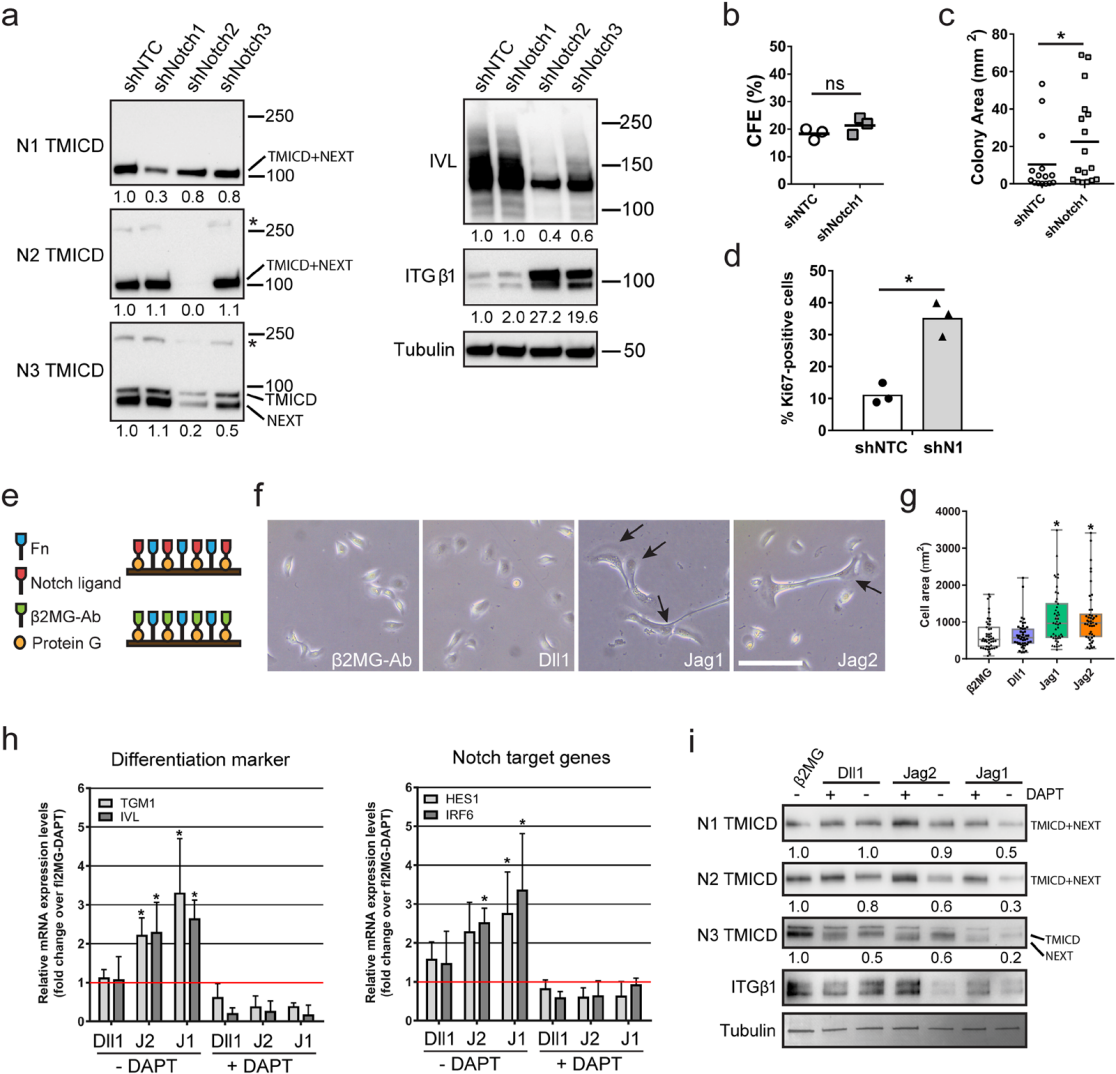
Distinct and overlapping roles of Notch receptors and ligands. (**a**) Western blots of epidermal sheets generated from keratinocytes (strain km) expressing either a non-targeting control shRNA (shNTC) or Notch receptor paralogue-silencing shRNAs (set 1, see Supplementary Table 5), using antibodies against the TMICD and NEXT fragments of Notch1, 2 or 3, or IVL and ITGβ1. Tubulin was used as loading control. Asterisks indicate the unprocessed precursor proteins also detected by Notch receptor antibodies. Numbers below lanes represent protein ratios relative to an arbitrary level of 1.0 set for control samples (shNTC). (**b**,**c**) Clonal growth assays of human keratinocytes (strain km) expressing the indicated shRNAs. (**b**) Colony formation efficiency (CFE). Data are from n = 3 independent experiments performed with three technical replicates. Individual data points represent average percentage of colonies formed per number of cells seeded; lines represent the mean. (**c**) Distribution of colony area (individual data points). Shown are the pooled data from the three independent experiments performed in (**b**); lines represent the mean. P-values were calculated using unpaired t test with Welsh’s correction. (*p < 0.05; ns, not significant). (**d**) Quantification of proliferation by human keratinocytes (strain km) expressing the indicated shRNAs. Data shown are from n = 3 independent experiments. Individual data points represent the percentage of Ki67-positive cells in each experiment; bars represent the mean. >500 cells were analysed per experiment. P-values were calculated using unpaired t test with Welsh’s correction (*p < 0.05). (**e**) Schematic illustration of the functionalized cell culture substrates used in (**f–i**). (**f**) Representative phase contrast images of sparse cells growing on cell culture substrates functionalized with the indicated proteins. Arrows indicate cells with elongated and flattened shapes. Bar, 100 µm. (**g**) Quantification of spread cell area. Box and whisker plots indicate the median (middle line in the box), the mean (small crosses), 25^th^ percentile (bottom line of the box), 75^th^ percentile (top line of the box), and the minimum and maximum (whiskers). P-values were calculated using one-way ANOVA with Holm Sidak’s multiple comparisons test (*p < 0.05). (**h**) Q-RT PCR analysis of mRNA levels of differentiation marker and Notch signalling target genes in cells (strain km) growing in the presence or absence of DAPT on substrates functionalized with the indicated proteins. Data shown are from n = 3 independent experiments. Individual data points represent fold change in mRNA abundance (normalized to 18sRNA) compared to control (cells growing on substrates functionalized with anti-β2MG antibodies and not treated with DAPT, red lines) in each experiment. Bars represent the means. Error bars represent S.D. P-values were calculated using one-way ANOVA with Holm Sidak’s multiple comparisons test (*p < 0.05). (**i**) Western blots of cells seeded in the presence or absence of DAPT on substrates functionalized with the indicated proteins, probed with antibodies, as indicated. Tubulin was used as loading control. Numbers below lanes are protein ratios relative to an arbitrary level of 1.0 set for control samples (cells growing on substrates functionalized with anti-β2MG antibodies and not treated with DAPT).

To test the functions of different Notch receptors, we silenced their expression in human keratinocytes using two sets of different shRNAs (Fig. 2a–d; Suppl. Fig. 1a,b; Suppl. Table 5), and allowed the cells to form stratified epidermal sheets^25,26^. We found that knockdown of Notch2 or Notch3 inhibited terminal differentiation, as evidenced by reduced expression of IVL and increased expression of ITGB1 (Fig. 2a, Suppl. Fig. 1a). The effect on terminal differentiation was even more pronounced using a shRNA that reduced the expression of Notch2 and Notch3 (Fig. 2a).

Knockdown of Notch1 led to only a small increase in ITGB1 expression and did not impair terminal differentiation (Fig. 2a, Suppl. Fig. 1a). However, keratinocytes expressing Notch1-targeting shRNAs formed larger colonies than cells expressing a non-targeting shRNA (Fig. 2b,c) and were more proliferative (Fig. 2d). There was also a reduction in abortive clones formed by shNotch1-expressing cells (Suppl. Table 1)^11,16^. Together, our findings indicate increased SC proliferation on Notch1 knockdown^27,28^. We conclude that Notch1 functions to reduce expansion of the epidermal SC compartment, as previously shown in the context of mouse epidermis^11,12,29^, while Notch2 and Notch3 cooperate to promote terminal differentiation.

We next examined the response of keratinocytes to different Notch ligands. To this end we functionalized cell culture surfaces with mixtures of fibronectin (FN) and immobilized recombinant Fc-tagged Notch ligands (Fig. 2e). This strategy allowed us to enrich for SC on the basis of rapid FN adhesion^22^, and measure differentiation and Notch target gene expression in the presence or absence of DAPT. Indirectly immobilized antibodies to the β2 microglobulin (β2MG) subunit of the major histocompatibility complex acted as control substrates (Fig. 2e)^30^. 24 hours after seeding, a fraction of single keratinocytes exposed to Jagged1 and Jagged2 displayed enlarged and elongated shapes typical of terminally differentiating cells^31^, while exposure to Dll1 ligands or anti-β2MG antibodies had no such effect (Fig. 2f,g). Q-RT PCR analysis confirmed the induction of differentiation marker (TGM1, IVL) expression by Jagged1 and Jagged2, correlating in each case with upregulation of the Notch signalling target genes HES1 and IRF6 (Fig. 2h). Since we did not test dose-responsiveness for the different Notch ligands, we cannot rule out the possibility that higher concentrations of recombinant Dll1 might also induce differentiation.

To examine differential activation of specific Notch receptors we exploited the fact that prolonged exposure to Notch ligand leads to reduced TMICD levels due to persistent S2 and S3 cleavage^24^. Under such conditions, parallel treatment with DAPT to block S3 cleavage causes accumulation of the NEXT fragment, thereby confirming that the observed reduction of TMICD levels is specifically caused by receptor activation^24^. Western blotting with antibodies detecting the TMICD and the NEXT fragment produced by S2 cleavage revealed that all Notch receptors were most potently activated by Jagged1 (Fig. 2i). ITGB1 was downregulated in response to Jagged1 and 2 but not Dll1. These findings confirm that engagement of Jagged1 and 2 with Notch receptors promotes terminal differentiation.

### Notch signalling can over-ride cell spreading to induce terminal differentiation

*In vitro*, terminal differentiation can be induced through distinct cellular signalling pathways, including—in addition to Notch signalling—serum stimulation, inhibition of EGFR signalling, stimulation with BMP2/7, and restricted cell spreading^32–34^. To examine the response of individual keratinocytes to the combined stimuli of cell spreading and Notch activation, we employed arrays of micropatterned 20 and 50 μm diameter circular adhesive islands to selectively capture single epidermal SCs^35^. Cells captured on the small adhesive islands do not spread and are stimulated to differentiate due to activation of serum response factor (SRF) and nuclear exclusion of YAP/TAZ downstream of actin polymerisation^32,34^.

**Figure 3:**
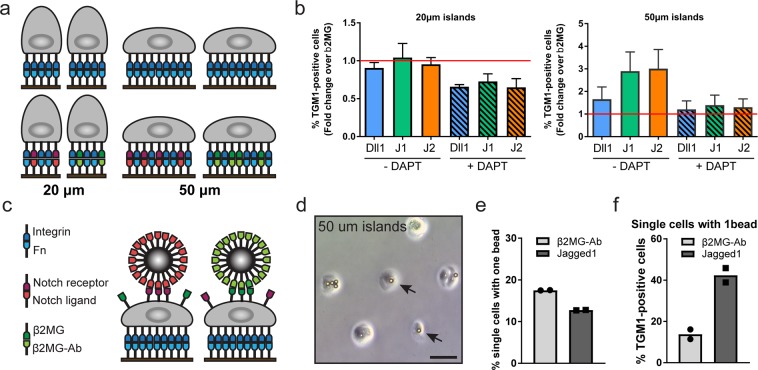
Notch signalling in spread and rounded cells. (**a**) Schematic illustration of strategy used to activate Notch signalling in single SCs by adherence to functionalised micro-patterned substrates. (**b**) Single cells (strain km) were captured on micro-patterend substrates functionalized with the indicated proteins and grown in the presence or absence of DAPT. Data shown are from n = 3 independent experiments. Individual data points represent fold change of % TGM1-positive cells relative to the control (cells growing on substrates functionalized with anti-β2MG antibodies and not treated with DAPT, red lines) in each experiment. Bars represent the mean. 400–800 single cells were analysed per experiment. P-values for pairwise comparisons were calculated using one-way ANOVA with Holm Sidak’s multiple comparisons test and are listed in Suppl. Table 2. (**c**) Schematic illustration of strategy used to activate Notch signalling in single SCs by exposure to functionalised microbeads. (**d–f**) SCs (strain km) were captured on 50 µm circular adhesive islands and exposed to microbeads functionalised with anti-β2MG antibodies or recombinant Jagged1. (**d**) Representative phase contrast image. Arrows indicate single cells with one attached bead. Bar, 50 µm. (**e**) Percentage of single cells with a single attached bead. > 300 cells were analysed per experiment. (**f**) Percentage of TGM1-positive single cells with a single attached bead. 60–75 single cells were analysed per experiment. Data shown are from n = 2 independent experiments (individual data points, bars represent the mean).

By functionalising the adhesive islands with mixtures of FN and indirectly immobilized Notch ligands we asked if exposure to a Notch signal could over-ride the anti-differentiation effect of cell spreading on large islands (Fig. 3a). Single cells undergoing terminal differentiation were identified using an automated high content imaging analysis pipeline (Suppl. Fig. 2a). While 48 hours of exposure to Jagged1 and Jagged2 ligands increased the frequency of single cells that differentiated on large islands, this was not the case for cells on small islands (Fig. 3b, Suppl. Fig. 2b, Suppl. Table 2). Consistent with previously published findings^34^, we observed a trend towards reduced numbers of differentiating cells on small islands upon DAPT treatment. However, overall, this trend was not statistically significant (Fig. 3b, Suppl. Fig. 2b, Suppl. Table 2).

To test if a localized Notch signal could induce differentiation, we exposed cells captured on 50 μm diameter adhesive islands to 10 μm diameter fluorescent microbeads functionalized with recombinant Jagged1 or anti-β2MG antibodies (Fig. 3c,d). To confirm that the two high-affinity protein interactions used to functionalise the beads (streptavidin–biotin and protein G–Fc-tag) resulted in presentation of the ligands in the appropriate conformation and density to induce the correct biological response at the cell surface, we performed pilot experiments using beads functionalized with recombinant E-cadherin^36^ (Suppl. Fig. 2c–e). We found that, as anticipated, such beads were captured by the cells in a calcium-dependent fashion (Suppl. Fig. 2d), and we observed clustering of endogenous α-catenin molecules at the bead-cell interface, indicating formation of adherens junctions^36^ (Suppl. Fig. 2e). To facilitate detection of single cells with a single attached bead, we also developed a semi-automated high content imaging analysis pipeline (Suppl. Fig. 2f). We found that, in comparison to beads functionalised with anti-β2MG antibodies, attachment of single Jagged1-functionalised beads led to a substantial increase in the numbers of cells that differentitated within 24 hours as assessed by TGM1 staining (Fig. 3e,f). These findings support a mechanism whereby a localised Notch signal is able to induce the terminal differentiation programme in single human epidermal SCs.

### Cis inhibition mediated by Dll1 inhibits Jagged-induced differentiation

**Figure 4:**
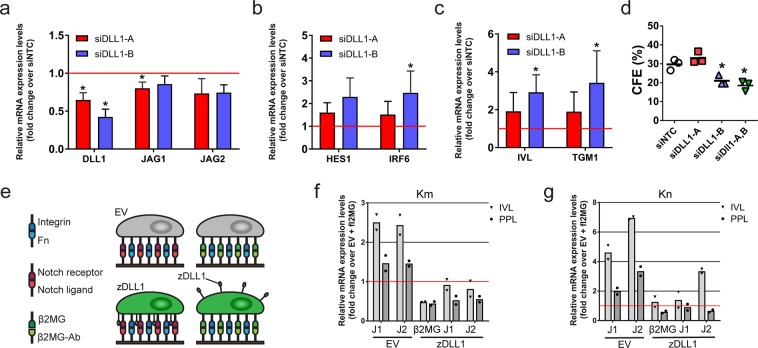
Cis inhibition mediated by Dll1 inhibits Jagged-induced differentiation. (**a–c**) Q-RT PCR analysis of mRNA levels of Notch ligands (**a**), Notch target genes (**b**) and terminal differentiation markers (**c**) in keratinocytes (strain km) transfected with two different Dll1-specific siRNAs or with a non-targeting control (NTC) siRNA and cultured under conditions that facilitated cell-cell interaction. Data shown are from n = 3 independent experiments performed with two biological replicates (independent siRNA transfections). Individual data points represent the average fold change in mRNA abundance (normalized to 18sRNA) compared to siNTC in each experiment. Bars represent the mean. P-values were calculated using one-way ANOVA with Holm Sidak’s multiple comparisons test (*p < 0.05). (**d**) Clonal growth of keratinocytes (strain km) transfected with the indicated siRNAs. Data shown are from n = 3 independent experiments performed with three technical replicates. Individual data points represent average percentage of colonies formed per number of cells seeded (colony formation efficiency, CFE); lines represent the mean. P-values were calculated using one-way ANOVA with Holm Sidak’s multiple comparisons test (*p < 0.05). (**e**) Schematic outline of the experimental setup used in (**f**,**g**). (**f**,**g**) Q-RT PCR analysis of terminal differentiation marker expression in keratinocytes (strain km, (**f**); strain kn, (**g**)) expressing zDLL1 or EV 48 hours after seeding onto substrates functionalized with the indicated proteins. Data shown in **f** and **g** are from n = 1 experiment performed with two technical replicates. Individual data points represent the average fold change in mRNA abundance (normalized to 18sRNA) compared to control (EV cells growing on substrates functionalized with anti-β2MG antibodies) in each experiment. Bars represent the means.

Our studies point to a different role for Dll1 compared to Jagged1 and Jagged2 in regulating exit from the SC compartment, consistent with earlier studies demonstrating that keratinocytes expressing high Dll1 levels induce differentiation in neighbouring cells with lower Dll1 levels^24,25^. To re-assess the function(s) of Dll1 in human keratinocytes, we knocked down endogenous expression by RNA interference. Although it is more difficult to silence the expression of weakly than highly expressed genes^37^, we were able to identify one siRNA that reduced the levels of Dll1 mRNA by over 50% without considerably affecting Jagged1 and 2 levels (Fig. 4a). Dll1 knock down led to a significant increase in expression of the Notch target gene IRF6 (Fig. 4b) and the differentiation markers IVL and TGM1 (Fig. 4c), as well as reduced colony formation (Fig. 4d). These results suggest that Dll1 has a cis-inhibitory function that renders Notch receptors on the surface of SCs non-responsive to ligand stimulation by neighbouring cells^17,38^.

To test whether such a mechanism was indeed operating in the human epidermal SC compartment, we overexpressed zebrafish Dll1(zDll1)^16^ in two different strains of human keratinocytes (Suppl. Fig. 3) and seeded cells at low cell density onto substrates functionalized with Jagged1 and Jagged2 (Fig. 4e). In this experimental setup, zDll1-mediated cis-inhibition of endogenous Notch receptors should block the response to Jagged1 and Jagged2. Consistent with this hypothesis, the Jagged1- and Jagged2-induced increase in IVL and PPL expression was prevented in cells overexpressing zDll1 (Fig. 4g,f). Thus, we conclude that a major function of Dll1 in the human IFE stem cell compartment is to cis-inhibit Notch signals from neighbouring cells.

## Discussion

It is well known that the Notch signalling pathway plays a central role in epidermal homeostasis and that Notch inactivation has a pro-tumorigenic effect in multilayered epithelia. However, the ways in which Notch executes these functions are highly complex and include cell autonomous and non-cell autonomous signalling within the epidermis, synergistic and antagonistic relationships with other signalling pathways, notably Wnt, Notch and YAP, and communication between the epidermis and cells of the immune system and stroma^8,39–41^. We have now used cultured human keratinocytes to map the changes in expression of Notch receptors and ligands associated with exit from the SC compartment and to perform functional assays of the effects of gene knockdown.

Our findings indicate that inactivation of Notch2 and Notch3 inhibit terminal differentiation, while Notch1 knockdown has a more pronounced effect on proliferation than terminal differentiation. Jagged1 and Jagged2 act as terminal differentiation stimuli, while Dll1 has the opposite effect. The ability of Jagged1 and Jagged2 to promote differentiation of spread keratinocytes on micropatterned islands leads us to speculate that Notch signalling may activate the SRF pathway and inactivate YAP/TAZ, providing an interesting counter-point to the observation that Notch and Hes1 are repressed in SRF knockout mouse epidermis^42^ and that knockdown of YAP/TAZ in human keratinocytes activates Notch signalling^34^. The concept of an interplay between Notch, SRF and YAP/TAZ signalling is supported by our finding that DAPT partially inhibits differentiation of rounded cells on micropatterned substrates.

The function of the Notch ligand Dll1 is particularly intriguing. Dll1 expression is patterned within the basal layer of fetal human and mouse epidermis^16,43^, and in human epidermis the distribution of Dll1 + basal layer clusters matches the distribution of stem cells^43^. In addition Dll1 is transcriptionally controlled in a mechano-sensitive manner by YAP and TAZ^34^. Single cell gene expression profiling indicates that in culture there are two SC subpopulations that differ in Dll1 expression, but not in expression of Notch pathway genes^44^. The Dll1 cytoplasmic domain binds syntenin and this association mediates stem cell clustering *in vitro*^16,45^. Knockdown of syntenin increases Dll1-dependent Notch activation^16^. Our studies provide experimental evidence for a recently proposed new function for Dll1 within stem cell clusters, namely preventing Notch signalling-mediated differentiation by cis-inhibiting Notch receptors from receiving signals in-trans^34^. It will now be of interest to examine how Dll1 expression affects the integration of signals involving Notch and additional pathways.

## Materials and Methods

### Human tissues

No human tissue was collected as part of this study. The human cells that we cultured for this study were isolated prior to 2004 and cryopreserved. Therefore, the tissue from which they were isolated is not covered by the UK Human Tissue Act (2004).

### Cell culture

Stock cultures of human keratinocytes (strains kn and km) from neonatal foreskin epidermis were used at passages 3–6. Each strain name refers to an individual foreskin. Primary cell cultures were established from surgically discarded neonatal foreskins as described^8–10^, expanded at passage 1 and then frozen down to prepare stock cultures. 3T3-J2 fibroblasts were originally obtained from Dr. James Rheinwald (Department of Dermatology, Harvard Skin Research Centre, USA) and were not authenticated. All cell stocks were routinely tested for mycoplasma contamination and were negative.

Human keratinocytes were cultured in complete FAD medium, containing 1 part Ham’s F12, 3 parts Dulbecco’s modified Eagle’s medium (DMEM), 10^−4^ M adenine, 10% (v/v) FBS, 0.5 μg ml^−1^ hydrocortisone, 5 μg ml^−1^ insulin, 10^−10^ M cholera toxin and 10 ng ml^−1^ EGF, on mitotically inactivated 3T3-J2 cells as described previously^22,25,35^. 3T3-J2 cells were cultured in high-glucose DMEM (Sigma-Aldrich) supplemented with 100 IU ml^−1^ penicillin, 100 μg ml^−1^ streptomycin and 10% (v/v) adult BS (Life Technologies)^35^. 3T3-J2 cells were mitotically inactivated by mitomycin C-treatment (3 h, 4 μg ml^−1^ final concentration, Sigma-Aldrich)^35^.

In some experiments human keratinocytes were seeded at a density of 3.5 × 10^4^ cells cm^−2^ in cell culture dishes (Falcon) coated with rat-tail collagen type I (20 µg ml^−1^ in PBS, BD Biosciences), grown to confluence in keratinocyte serum-free medium (KSFM) containing 30 µg ml^−1^ bovine pituitary extract and 0.2 ng ml^−1^ EGF (Thermo Fisher Scientific) and then switched to complete FAD medium to induce terminal differentiation and stratification^35^. For experiments using glass micro-chips containing micro-patterned arrays of circular islands, pre-confluent human keratinocytes were gently disaggregated in trypsin/EDTA following removal of the feeder layer, filtered twice through a 40 µm cell strainer (Falcon) and re-seeded onto the substrates at a density of 1 × 10^4^ cells cm^−2^ in KSFM supplemented with 10 µM of the γ-secretase inhibitor DAPT. DAPT was obtained from Sigma-Aldrich (D5942) and dissolved in sterile DMSO to obtain a 20 mM stock solution. To prevent auto-activation of Notch receptors due to chelation of Ca^++^ ions^46^, DAPT (10 µM) was also present during feeder layer removal and trypsinisation. Cells were allowed to adhere for 2 h and the substrates were then rinsed five times with fresh medium to remove non-attached cells^35^. Cells were subsequently cultured in complete FAD medium without DAPT for up to 48 hours.

Cell fractions enriched for human epidermal stem cells, committed progenitors and terminally differentiated cells were prepared as described^22,35^ by adhesion to human placenta collagen type IV (Sigma-Aldrich). In some experiments cells were transduced with an empty vector (EV) or a zDll1 retroviral vector, as described previously^16^. Expression of the zDll1 transgene was validated by immunofluorescence microscopy using zDll1-specific antibodies^16^.

### Clonal growth assays

100 live (Trypan Blue-negative) human keratinocytes were seeded per condition into triplicate wells (containing a 3T3-J2 feeder layer) of a 6-well dish (Falcon). After 12 days, feeder cells were removed by rinsing with EDTA and colonies were either fixed in 4% (w/v) paraformaldehyde for 10 min and stained with 1% Rhodanile Blue (1:1 mixture of Rhodamine B and Nile Blue A (Acros Organics))^22^, or simultaneously fixed and stained with Crystal Violet solution (0.4% (w/v) crystal violet, 20% (v/v) methanol)^35^. Colonies were imaged and counted using an automated cell colony counter (Gelcount™, Oxford Optronix, UK), and colony forming efficiency (CFE) was calculated as the average percentage of seeded cells that formed colonies^35^. Colony area was measured using the Fiji image processing software package and the “Analyze Particles” tools, with a minimum particle size of 0.01 mm^2^ ^35^. Colonies were scored as abortive if they contained fewer than 40 cells, the majority of the cells being large and terminally differentiated^43^.

### Microfabrication

CYTOOchips™ custom-manufactured composite micropatterned glass slides containing arrays of 20 and 50 µm diameter circular islands were purchased from CYTOO (Grenoble, France)^35^. The custom-designed template masks to print new slides are available upon request (Custom_CC20_Q13-24-38).

### Preparation of functionalised substrates

6-well plates (Falcon) were first coated with a mixture of recombinant protein G (4 µg/cm^2^) (Invitrogen) and human fibronectin (4 µg/cm^2^) (Corning) diluted in PBS over night (o/n) at room temperature (RT). Unbound proteins were removed by washing the plates three times with PBS, followed by blocking with 1% (w/v) BSA in PBS at RT for 1 hour. Wells were incubated for 4 h at RT with recombinant Fc-tagged Notch ligand proteins Dll1 (Adipogen Life Science), Jagged1 and Jagged2 (R&D) or antibodies against the β2 microglobulin (β2MG) subunit of the human major histocompatibility complex (Abcam) (2.5 µg/cm^2^), diluted in 0.1% (w/v) BSA in Hanks Buffered Salt Solution (HBSS). Any protein not bound to protein G was removed by washing the substrates three times with HBSS. Glass microchips containing micropatterned arrays of 20 μm and 50 μm circular islands were placed into 6-well plates (Falcon) and functionalised as described above. Functionalised substrates were prepared fresh for each experiment and kept in HBSS until use.

### Preparation of functionalised microbeads

200 µl (~3.4 × 10^6^ beads) ProActive FlashRed-fluorescent (excitation wavelength 660 nm, emission wavelength 690 nm) streptavidin-coated polystyrene microbeads (SuperAvidin™ Microspheres, Bangs Laboratories, mean diameter 10.14 µm) were resuspended by vortexing (30 sec), followed by sonication in a waterbath (Camsonix C-Series Heated Ultrasonic Cleaning Baths, Camlabs) for 30 sec at RT. Beads were then pelleted by centrifugation (1,200xg for 15 min at RT) and washed twice with 1 ml PBS. Beads were resuspended in 500 µl PBS containing 100 µg ml^−1^ biotinylated protein G (Sigma), and incubated overnight with constant rotation at 4 °C. After two cycles of centrifugation and resuspension in PBS, beads were resuspended in 200 µl HBSS and added to 200 µl HBSS containing either 20 µg recombinant Fc-tagged Jagged1 (R&D) or 20 µg of antibodies against the β2 microglobulin (β2MG) (Abcam), and incubated overnight with constant rotation at 4 °C. Beads were then washed twice in HBSS and blocked by subsequent incubations with 15 mM biotin (Sigma) and 1% (w/v) BSA in HBSS (1 h each) with constant rotation at RT. After two washing cycles in HBSS, beads were added to cells captured on glass micro-chips containing micro-patterned arrays of 20 and/or 50 µm diameter circular islands. After a 3 h incubation period, non-cell-attached beads were washed away, and cells were cultured for further 24 h.

### RNA extraction and quantitative real time PCR

Total RNA was isolated from cultured cells using the RNeasy kit (Qiagen)^35^. Complementary DNA was generated using the QuantiTect Reverse Transcription kit (Qiagen). Q-RT PCR analysis of cDNA was performed using either qPCR primers (published or designed with Primer3) and Fast SYBR green Master Mix (Life Technologies), or Taqman probes and TaqMan Fast Universal PCR Master Mix (Thermo Fisher Scientific)^35^. RT-qPCR reactions were run on the CFX384 Real-Time System (Bio-Rad)^35^. 18S rRNA, GAPDH and TBP were used as housekeeping genes for normalization^35^. Please refer to Supplementary Table 3 for qPCR oligo sequences and Taqman probes.

### siRNA transfection

siRNA nucleofection was performed with the Amaxa 96-well shuttle system (Lonza)^35^. Pre-confluent human keratinocyte cultures were disaggregated and re-suspended in cell line buffer SF (Lonza). For each 20 μl transfection (program FF-113) reaction, 2 × 10^5^ cells were mixed with 1 μM siRNA duplexes as described previously^33,35^. Transfected cells were allowed to recover at ambient temperature for 10 min and were subsequently re-plated onto rat-tail type I collagen (20 µg ml^−1^ in PBS, BD Biosciences)-coated cell culture well plates (Falcon) and grown in KSFM for 24 h before being used for downstream assays^35^. 27mer siRNA oligo duplexes (SR509346, Origene) were used for gene knockdown of human DLL1 (the sequences of siRNA oligos can be found in Supplementary Table 4). Non-targeting control siRNAs were from Ambion (AM4611 and AM4637).

### shRNA-mediated gene silencing

Human keratinocytes, cultured on feeder cells to ~70% confluence, were disaggregated in trypsin/EDTA and cells (5 × 10^5^) were seeded into 6-well cell culture plates (Falcon) coated with rat-tail collagen type I (20 µg ml^−1^ in PBS, BD Biosciences), and cultured for 24 h in KSFM medium^35^. Cells were then infected with MISSION® lentiviral particles (Sigma-Aldrich, the sequences of the shRNAs can be found in Supplementary Table 5) at a MOI of 3 in the presence of 5 µg ml^−1^ polybrene (Sigma-Aldrich)^35^. Medium was replaced after 24 h and shRNA-expressing cells were selected for 72 h using puromycin (2 µg ml^−1^, Sigma-Aldrich)^35^.

### Immunofluorescence microscopy of cultured cells

Cultured cells grown on CYTOO microchips or on glass coverslips (coated with rat-tail type I collagen (20 µg ml^−1^ in PBS, BD Biosciences) were fixed in 4% (w/v) paraformaldehyde (Sigma) for 10 min and permeabilized with 0.2% (v/v) Triton X-100 for 5 min at ambient temperature^35^. Human keratinocyte colonies grown in the presence of a 3T3-J2 feeder layer were fixed in 4% (w/v) paraformaldehyde (Sigma) for 45 min and permeabilized with 0.5% (v/v) Triton X-100 for 45 min at ambient temperature^35^. Samples were blocked for 1 h in 10% (v/v) FBS plus 0.25% (v/v) fish skin gelatin (Sigma-Aldrich) in 1x PBS (blocking buffer), and incubated with primary antibodies (diluted in blocking buffer) in a humid chamber overnight at 4 °C^35^. After washing with PBS, samples were incubated with Alexa Fluor^®^-conjugated secondary antibodies for 2 h at room temperature^35^. Primary antibodies are listed in Supplementary Table 6. Rhodamine–phalloidin (Thermo Fisher Scientific) was included in the secondary antibody solution where indicated to stain filamentous actin. Fixed and stained coverslips were mounted on glass slides with ProLong^®^ Gold anti-fade reagent containing DAPI (Thermo Fisher Scientific)^35^. Confocal images were acquired with a Nikon A1r point scanning confocal fluorescence microscope equipped with a Plan Apo VC 20x DIC N2 objective (Nikon) and controlled by NIS elements C software. Digital images were processed using NIS elements Advanced Research and Adobe software packages.

### High content imaging analysis

Cells were processed for immunofluorescence microscopy as described above. Automated image acquisition was performed on an Operetta^®^ high content imaging system (Perkin Elmer)^35^. For imaging, the entire CYTOO chip area was scanned; fields without fluorescence artefacts were selected, and z-stack images (z dimension 1 µm) were acquired using 10x long WD objectives via the DAPI (50 ms exposure), Alexa-488 (200 ms exposure) and Alexa-594 (200 ms exposure) channels^35^. Images were analysed using custom algorithms in the Harmony^®^ high-content analysis software package (Perkin-Elmer)^35^ (Supplementary Fig. 2a,f). For all image analyses, cells were initially defined using the DAPI channel, then the cytoplasm was segmented using one of the Alexa channels. Single cells and cell clusters were identified and separated using linear classifiers defined through PhenoLOGIC machine learning within Harmony^®^ software. Terminally differentiating cells were identified based on the presence of TGM1 fluoresence signals after background correction. The complete Harmony^®^ image analysis sequence is available on request.

### Western blotting

Cells were lysed on ice for 30 min in 1x RIPA buffer (Cell Signalling Technology) supplemented with PhosSTOP^®^ Phosphatase Inhibitor and cOmplete^®^ EDTA-free Protease Inhibitor Cocktails (Roche), and RIPA-soluble and -insoluble proteins were separated via centrifugation (16,000 × *g* for 20 min at 4 °C)^35^. The amount of total protein was quantified in RIPA extracts using the BCA kit (Pierce). Equivalent quantities of RIPA-solubilized proteins were resolved by SDS-PAGE in 4–20% Criterion TGX Stain-Free Precast Gels and transferred to Immun-Blot^®^ Low Fluorescence PVDF membranes (Bio-Rad Laboratories) using the Trans-Blot^®^ Turbo transfer system (Bio-Rad Laboratories)^35^. Protein transfer and equal protein loading were confirmed by enhanced tryptophan fluorescence imaging of PVDF membranes (Bio-Rad Laboratories)^35^. Membranes were blocked with 5% (w/v) non-fat milk supplemented with 0.05% (v/v) Tween-20 (PBS-T) and then probed with the indicated antibodies diluted in blocking buffer. Primary antibodies are listed in Supplementary Table 6. Primary antibody-probed blots were visualized with appropriate horseradish peroxidase-coupled secondary antibodies (Jackson ImmmunoResearch) using enhanced chemiluminescence (Clarity™ Western ECL, Bio-Rad Laboratories) according to the manufacturer’s instructions^35^. Protein bands were detected using a ChemiDoc Touch Imaging System (Bio-Rad Laboratories)^35^. Processing of western blot images was performed using Image Lab software (Bio-Rad Laboratories)^35^. For quantification of band intensities, exposures within the dynamic range were chosen^35^. Images of uncropped blots are shown in Supplementary Fig. 4.

### Microarray dataset analysis

Computational analysis of gene expression datasets was performed as described using microarray datasets obtained from human keratinocytes undergoing suspension-induced terminal differentiation^20^ (GEO databank GSE73147). We performed pairwise comparison between 0 h and 4, 8 and 12 h, and between 0 h and 4 h, 4 h and 8 h, and 8 h and 12 h. Heatmaps were generated using opensource Multiple Experiment Viewer (MeV_4_8) software.

### Reproducibility of experiments

Reproducibility of experiments was evaluated as follows. For fractionation of human keratinocyte cultures, 5 independent experiments were performed using independent cell stocks. Experiments involving micropatterned substrates were performed independently three times (using independent cell stocks and freshly functionalised substrates). Experiments involving shRNA treatments were performed with two different sets of shRNAs in two different strains of human keratinocytes, with comparable results. For clonal growth assays 2–3 independent experiments were performed with 2–3 technical replicates per condition. For western blotting experiments, representative blots from one of two experiments are shown. For immunostaining, representative images from one of two experiments are shown. Q-RT PCR analysis was performed on four technical replicates. For cis-inhibtion of Notch signalling, we perfomed two independent experiments using two different strains of human keratinocytes, independently infected with zDll1-expressing retrovirus, with two technical replicates per experiment.

### Statistics and graph generation

No statistical method was used to predetermine sample size. Statistical tests used to determine p values are specified in Figure Legends. All graphs were generated using GraphPad Prism 7.

### Antibodies

Primary antibodies are listed in Supplementary Table 6.

## Supplementary information

Supplementary information

## Data Availability

The authors declare that all data supporting the findings of this study are available within the paper and its Supplementary Information Files. There are no restrictions on data availability.
