# Supplementary material for: Delta-like 1-mediated cis-inhibition of Jagged1/2 signalling inhibits differentiation of human epidermal cells in culture
Source: Sci Rep. 2019 Jul 25;9:10825. doi: 10.1038/s41598-019-47232-2 (PMC6658703; doi:10.1038/s41598-019-47232-2)
Supplement: Supplementary file 1 — Supplementary information [file 41598_2019_47232_MOESM1_ESM.pdf]

Delta-like 1-mediated cis-inhibition of Jagged1/2 signalling inhibits differentiation of human epidermal cells in culture

Victor A. Negri[1], Meike Logtenberg[1,2], Lisa M. Renz[1,3], Bénédicte Oules[1], Gernot Walko[1,4,*,†], and Fiona M. Watt[1,*,†]

[1]Centre for Stem Cells and Regenerative Medicine, Faculty of Life Sciences & Medicine, King's College London, 28th Floor, Tower Wing, Guy's Hospital, SE1 9RT London, UK.
[2]Current address: Division of Immunology, The Netherlands Cancer Institute, Postbus 90203, 1006 BE Amsterdam, The Netherlands.
[3]Current address: Research Institute for Applied Bioanalytics and Drug Development, IMC University of Applied Sciences, A-3500 Krems an der Donau, Austria.
[4]Current address: Department of Biology and Biochemistry, University of Bath, Claverton Down, Bath BA2 7AY, United Kingdom
[*]Corresponding author. (email: g.walko@bath.ac.uk)
[*]Corresponding author. (email: fiona.watt@kcl.ac.uk)
[†]These authors contributed equally to this work.

Negri et al. Supplementary Information

Supplementary Figure 1 Validation of shRNA-mediated gene silencing of Notch receptor paralogues. (**a**, **b**) Keratinocytes (strain kn) expressing either a non-targeting control shRNA (shNTC), or Notch receptor paralogue-specific shRNAs (set 2, see Supplementary Table 5) were cultured under conditions enabling formation of epidermal sheets (see Materials and Methods). (**a**) Western blot analysis using antibodies against the TMICD and NEXT fragment of Notch1, 2 or 3, or ITGβ1. Tubulin was used as loading control. Asterisks indicate the unprocessed precursor proteins also detected by Notch receptor antibodies. Numbers below lanes are protein ratios relative to an arbitrary level of 1.0 set for control samples (shNTC). (**b**) Q-RT PCR analysis of Notch receptor mRNA levels. Data shown are from n = 1 experiment performed with n = 3 biological replicates (independent lentiviral infections). Individual data points represent the fold change in mRNA abundance (normalized to the mean of 18sRNA and TBP) compared to shNTC in each experiment. Bars represent the means.

Supplementary Figure 2 Image analysis workflows and validation of microbead functionalisation strategy. (**a**) Image analysis pipeline in Harmony software for identification of single differentiating cells. Input images (1) are segmented to identify nuclei (2) and image regions occupied by cells (3). Single cells and cell clusters are recognised using linear classifiers defined through PhenoLOGIC machine learning (4). Border objects and image artefacts are discarded via morphology and intensity assessment on nuclei and on cells (5). Single cells undergoing terminal differentiation are identified based on TGM1 fluorescence over threshold in the single cell population (6). (**b**) Percentage of terminally differentiating (TGM1-positive) cells in the experiments shown in Fig. 3b. Bars represent the means from n = 3 independent experiments. Error bars represent S.D. (**c**) Representative images of FlashRed-labelled microbeads functionalised with recombinant E-cadherin molecules and immunolabelled with anti-E-cadherin antibodies. Scale bar, 50 μm. (**d**) Quantification of microbead attachment to single cells. Cells were captured on micro-patterend substrates and incubated for 24 hours with microbeads (functionalised with recombinant E-cadherin) suspended in culture medium with high (1.8 mM) or low (0.05 mM) concentrations of $CaCl_2$. Data shown are from n = 1 experiment performed with n = 2 biological replicates (independent bead incubations on separate microchips). Individual data points represent the percentage of

cells with attached microbeads in each experiment. Bars represent the means. (**e**) Representative image (maximum intensity projection) of keratinocytes with attached microbeads (functionalised with recombinant Fc-tagged E-cadherin molecules), immunolabelled with antibodies against α-catenin and counterstained with phalloidin to label filamentous actin. Arrows indicate clustering of endogenous α-catenin molecules at the microbead-cell interface, demonstrating adherens junction formation. Scale bar, 50 µm. (**f**) Image analysis pipeline in Harmony software for identification of single cells with attached beads. Input images (1) are segmented to identify nuclei (2) and image regions occupied by cells (3). Single cells and cell clusters are recognised using linear classifiers defined through PhenoLOGIC machine learning (4). Border objects and image artefacts are discarded via morphology and intensity assessment on nuclei and on cells (5, 6). Fluorescent microbeads are detected using the spot identification module (7). Single cells with attached beads are identified based on bead fluorescence over threshold in the single cell population (8).

Supplementary Figure 3 Overexpression of zDLL1 in keratinocytes. (**a**) Representative images (maximum intensity projections) of keratinocytes (strain km) stably expressing zebrafish Dll1 (zDll1) or the empty vector (EV), immunolabelled with antibodies against zDll1 and counterstained with DAPI to reveal nuclei. Arrowheads, localisation of zDll1 at areas of cell-cell contact; asterisks, vesicular localisation of zDll1[32]. Scale bar, 100 µm. (**b**) Q-RT PCR analysis of mRNA levels of endogenous (hDll1) and zebrafish (zDll1) Dll1 in keratinocytes (experiment 1: strain km, experiment 2: strain kn; see Fig. 3f, g) expressing zDLL1 or EV. Data shown are from n = 2 technical replicates. Individual data points represent the mean ΔCq expression (normalized to the mean of GAPDH and TBP). Bars represent the means.

Supplementary Figure 4 Uncropped western blots.

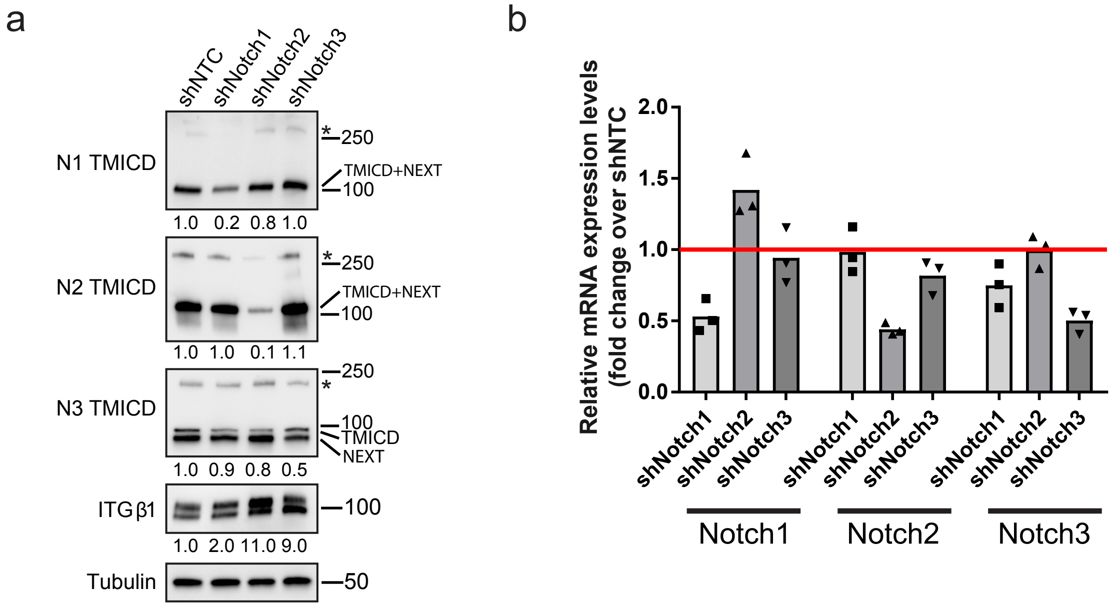

Negri et al. Fig.S1

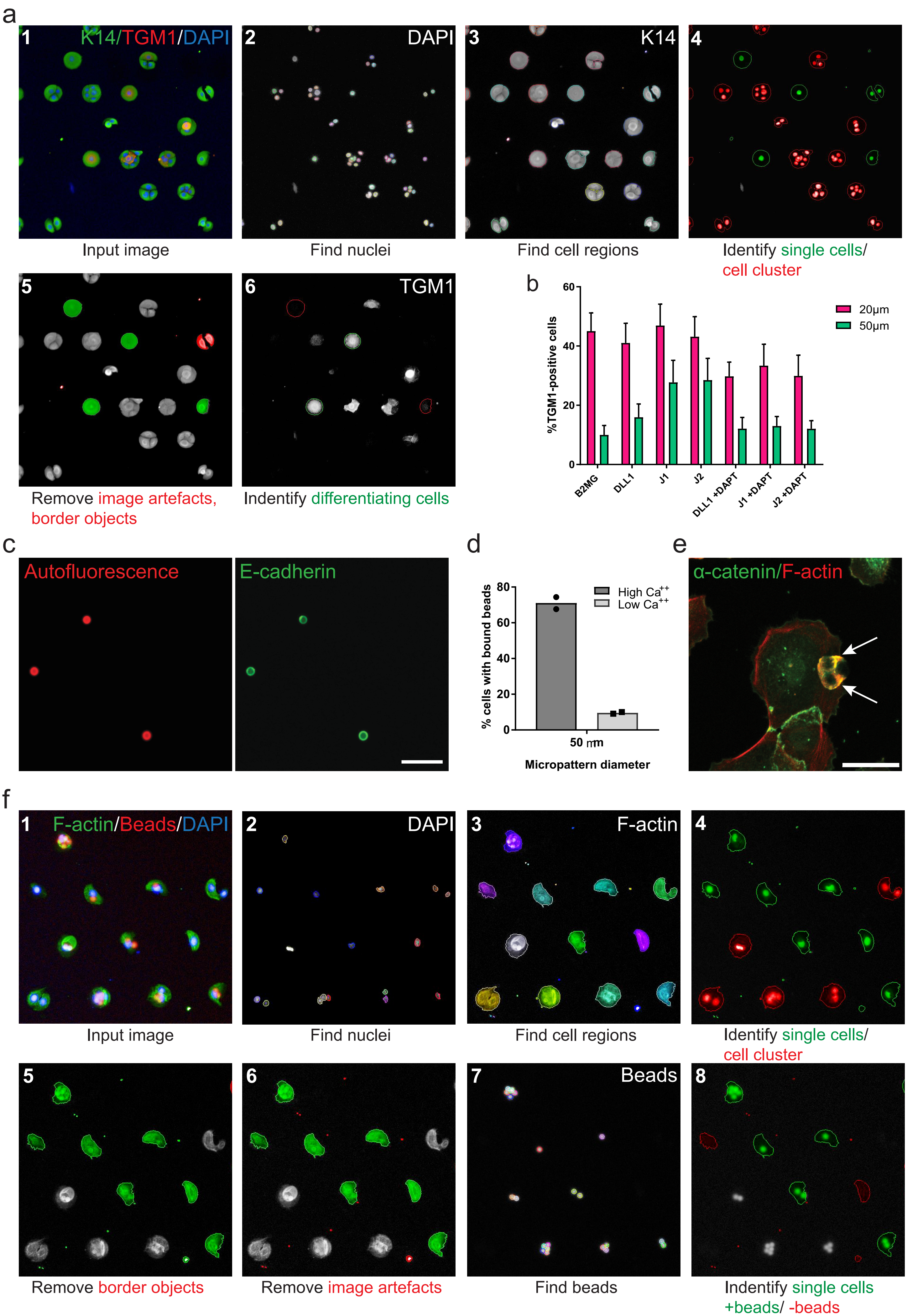

Negri et al. Fig.S2

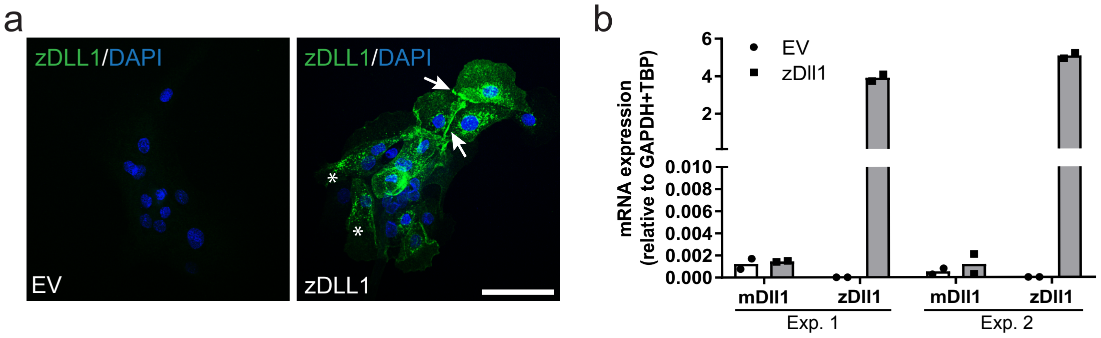

Negri et al. Fig.S3

Fig 1i

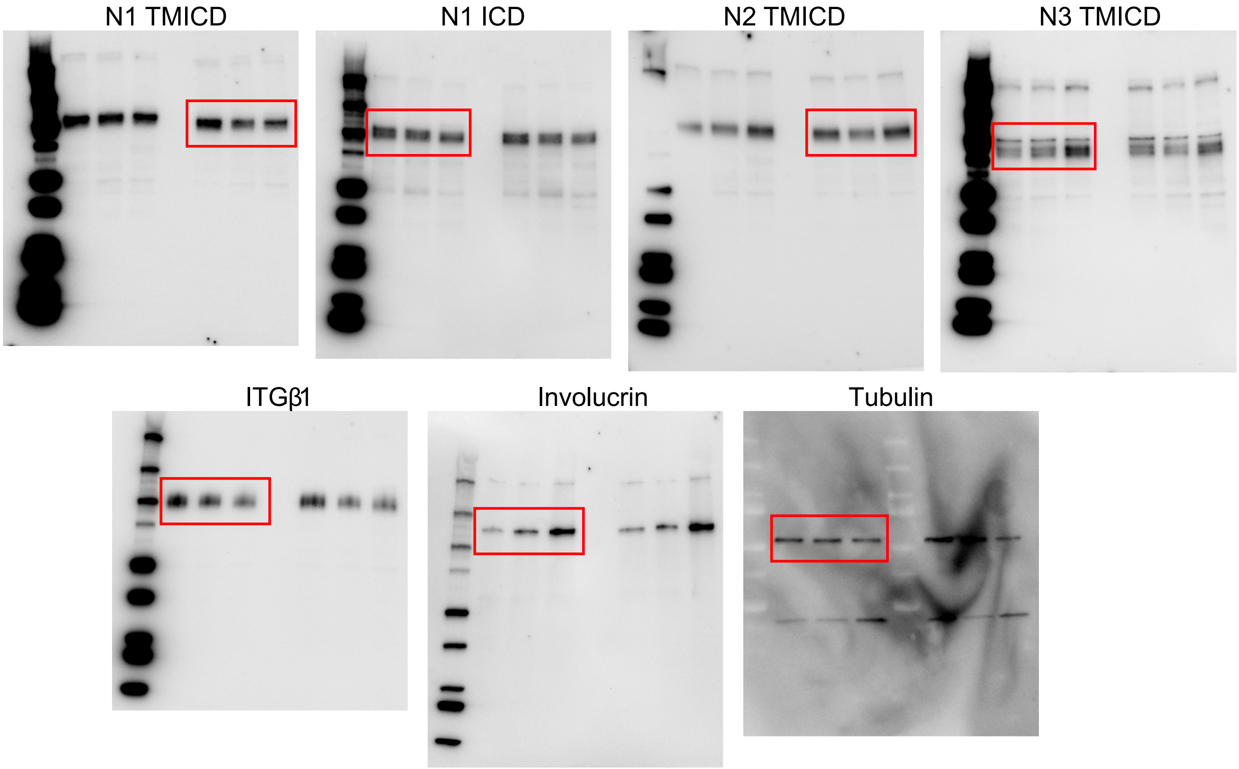

Fig 1j

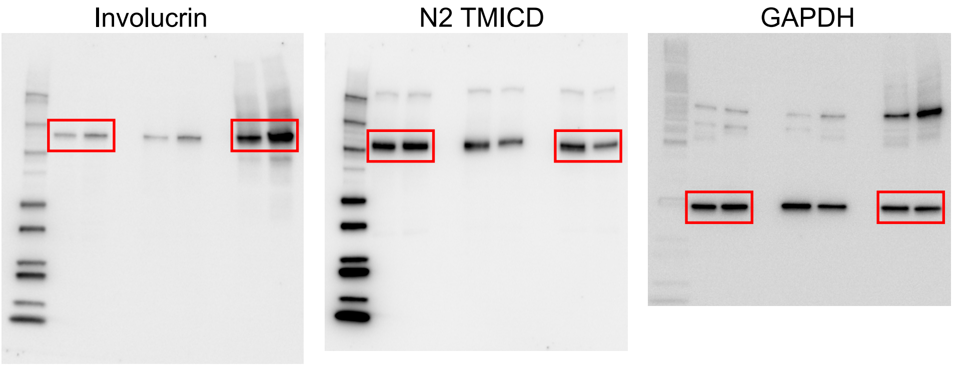

Fig. 2a

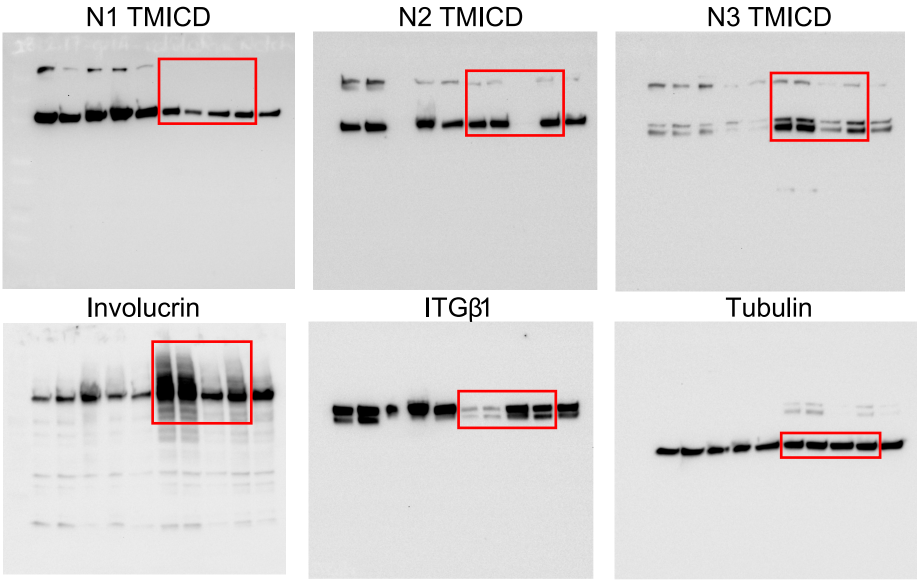

Fig 2i

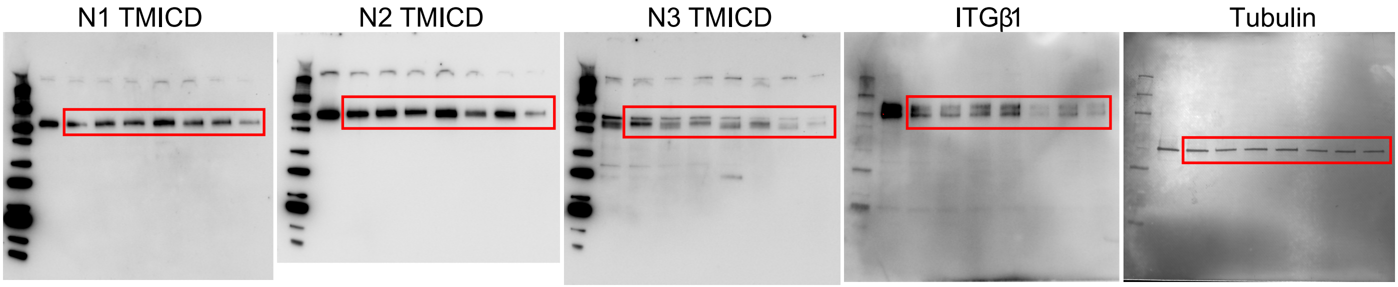

Fig S1a

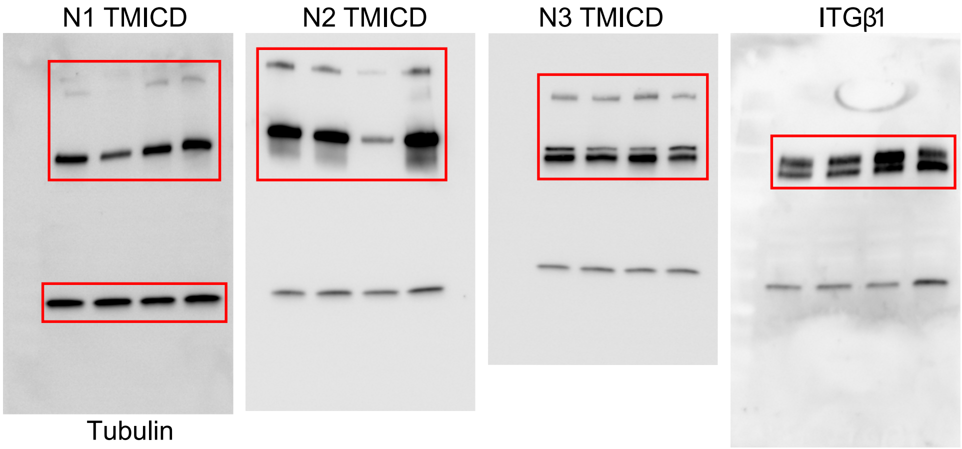

Supplementary Table 1

Effect of Notch1 knockdown on clonal growth of keratinocytes.

Keratinocytes (strain km) were transduced with the indicated shRNAs and cultured on a fibroblast feeder layer. Data are the mean ± S.D. from three experiments.

shRNA	% abortive clones	S.D.
shNTC	83.8	7.65
shNotch1	57.4	8.51

Supplementary Table 2: **Holm-Sidak's multiple comparisons test corresponding to Figure 3b**

	**20 um islands**				**50 um islands**			
Pairwise comparison	Mean Diff.	Significant?	Summary	Adj. P Value	Mean Diff.	Significant?	Summary	Adj. P Value
Ctrl vs. DLL1 +DAPT	0.343	**Yes**	*	0.0178	-0.1386	No	ns	0.9975
Ctrl vs. DLL1 -DAPT	0.09616	No	ns	0.9146	-0.5571	No	ns	0.919
Ctrl vs. J1 +DAPT	0.2726	No	ns	0.0721	-0.392	No	ns	0.9831
Ctrl vs. J1 -DAPT	-0.04238	No	ns	0.9636	-1.899	**Yes**	**	0.0092
Ctrl vs. J2 +DAPT	0.3505	**Yes**	*	0.0157	-0.3032	No	ns	0.9947
Ctrl vs. J2 -DAPT	0.04715	No	ns	0.9636	-2.005	**Yes**	**	0.006
DLL1 +DAPT vs. DLL1 -DAPT	-0.2468	No	ns	0.1127	-0.4185	No	ns	0.9829
DLL1 +DAPT vs. J1 +DAPT	-0.07032	No	ns	0.9333	-0.2534	No	ns	0.9965
DLL1 +DAPT vs. J1 -DAPT	-0.3853	**Yes**	**	0.0074	-1.76	**Yes**	*	0.0157
DLL1 +DAPT vs. J2 +DAPT	0.007503	No	ns	0.9636	-0.1646	No	ns	0.9975
DLL1 +DAPT vs. J2 -DAPT	-0.2958	**Yes**	*	0.0449	-1.867	**Yes**	*	0.0101
DLL1 -DAPT vs. J1 +DAPT	0.1765	No	ns	0.4089	0.1651	No	ns	0.9975
DLL1 -DAPT vs. J1 -DAPT	-0.1385	No	ns	0.6716	-1.342	No	ns	0.0733
DLL1 -DAPT vs. J2 +DAPT	0.2543	No	ns	0.1025	0.2538	No	ns	0.9965
DLL1 -DAPT vs. J2 -DAPT	-0.04902	No	ns	0.9636	-1.448	**Yes**	*	0.0484
J1 +DAPT vs. J1 -DAPT	-0.315	**Yes**	*	0.0323	-1.507	**Yes**	*	0.0396
J1 +DAPT vs. J2 +DAPT	0.07782	No	ns	0.9333	0.08875	No	ns	0.9975
J1 +DAPT vs. J2 -DAPT	-0.2255	No	ns	0.1669	-1.613	**Yes**	*	0.0275
J1 -DAPT vs. J2 +DAPT	0.3928	**Yes**	**	0.0066	1.596	**Yes**	*	0.0281
J1 -DAPT vs. J2 -DAPT	0.08953	No	ns	0.9158	-0.1064	No	ns	0.9975
J2 +DAPT vs. J2 -DAPT	-0.3033	**Yes**	*	0.0401	-1.702	**Yes**	*	0.0194

Supplementary Table 3: List of qPCR primers and Taqman probes

qPCR primer

Gene	Forward primer	Reverse primer
18sRNA	GCAATTATTCCCCATGAACG	GGCCTCACTAAACCATCCAA
TBP	GTGACCCAGCATCACTGTTTC	GAGCATCTCCAGCACACTCT
HES1	TCAACACGACACCGGATAAAC	GCCGCGAGCTATCTTTCTTC
HEY1	GTTCGGCTCTAGGTTCCATGT	CGTCGGCGCTTCTCAATTATTC
IRF6	GCTCTCTCCCAATGACCTGGA	CCATGACGTCCAGCAGCTTGCTA
TGM1	GTTGCCCTTTGACCCCCGCA	CCCCGTGGTCAAACTGGCCG
IVL	GCCTCAGCCTTACTGTGAGT	TGTTTCATTTGCTCCTGATGG
PPL	GCAGAGTGACCTGGCTCGGCT	GCCGCATCCGCCTCTAGCAC
Notch1	TCCACCAGTTTGAATGGTCA	AGCTCATCATCTGGGACAGG
Notch2	GATCACCCGAATGGCTATGAAT	GGGGTCACAGTTGTCAATGTT
Notch3	TGGCGACCTCACTTACGACT	CACTGGCAGTTATAGGTGTTGAC
Notch4	TGTGAACGTGATGTCAACGAG	ACAGTCTGGGCCTATGAAACC

Taqman probes

Gene	Code
18sRNA	Hs03003631_g1
GAPDH	Hs02786624_g1
Jagged 1	Hs01070032_m1
Jagged 2	Hs00171432_m1
Dll1	Hs00194509_m1
Dll3	Hs01085096_m1
Dll4	Hs00184092_m1

Supplementary Table 4: List of siRNAs

Catalog Number	Gene Symbol	RefSeq	Sequence
SR309129	Dll1	NM_005618, XM_005266934	CGCAGAUCAAGAACACCAACAAGAA
SR309129	Dll1	NM_005618, XM_005266934	UGAACUGAAUUACGCAUAAGAAGCA

Supplementary Table 5: List of MISSION shRNA lentiviral transduction particles

shRNA set	Product Number	TRC Number	Gene target	Clone ID	Sequence
2	SHCLNV-NM_017617	TRCN0000	Notch1	NM_017617.x-903s1c1	CCGGGATGCCAAATGCCTGCCAGAACTCGAGTTCTGGCAGGCATTTGGCATCTTTTT
1	SHCLNV-NM_017617	TRCN0000	Notch1	NM_017617.3-6258s21c1	CCGGCCGGGACATCACGGATCATATCTCGAGATATGATCCGTGATGTCCCGGTTTTTG
2	SHCLNV-NM_024408	TRCN0000	Notch2	NM_024408.2-6334s21c1	CCGGCAAGATCCTGTTAGACCATTTCTCGAGAAATGGTCTAACAGGATCTTGTTTTTG
1	SHCLNV-NM_024408	TRCN0000	Notch2	NM_024408.2-6747s21c1	CCGGCCACATCCTCTCCAATGATTACTCGAGTAATCATTGGAGAGGATGTGGTTTTTG
2	SHCLNV-NM_000435	TRCN0000	Notch3	NM_000435.1-1431s1c1	CCGGCCAGTTCACCTGTATCTGTATCTCGAGATACAGATACAGGTGAACTGGTTTTT
1	SHCLNV-NM_000435	TRCN0000	Notch3	NM_000435.2-1524s21c1	CCGGTCTGCAAGGACCGAGTCAATGCTCGAGCATTGACTCGGTCCTTGCAGATTTTTG

Supplementary Table 6: List of antibodies

Antibody information					Dilution	
**Antibody**	**Company/Source**	**Catalog Number/Reference**	**Clone number**	**Description**	**Western Blot**	**IFM-cells**
Notch1	Cell Signaling Technology	3608	D1E11	Rabbit monoclonal	1:1000	
Cleaved Notch1 (Val1744)	Cell Signaling Technology	4147	D3B8	Rabbit monoclonal	1:1000	
Notch2	Cell Signaling Technology	5732	D76A6	Rabbit monoclonal	1:1000	
Notch3	Cell Signaling Technology	5276	D11B8	Rabbit polyclonal	1:1000	
Dll1		S Estrach et al., J Cell Sci (2007), 120(16):2944-52	Zdd2	Mouse monoclonal		1:500
anti-Integrin β1	Cell Signaling Technology	4706		Rabbit polyclonal	1:1000	
anti-Involucrin		D Hudson et al., Hybridoma (1992), 11(3):367-379	SY7	Mouse monoclonal	1:1000	1:1500
anti-Ki67	Cell Signaling Technology	9449	8D5	Mouse monoclonal		1:800
anti-α-Tubulin	Sigma-Aldrich	T6199	DM1A	Mouse monoclonal	1:2000	
anti-Keratin 14	Covance	PRB-155P		Rabbit polyclonal	1:1000	1:2000
anti-TGM1		Thacher SM and Rice RH, Cell (1985), 40:685-695	BC.1	Mouse monoclonal		1:1500
anti-GADPH	Millipore	MAB374	6C5	Mouse monoclonal	1:1000	
anit-E-cadherin		Shimoyama et al., Cancer Res (1989), 49:2128-2133	HECD-1	Mouse monoclonal		1:500
anti-alpha-Catenin	Cell Signaling Technology	3236		Rabbit polyclonal		1:200
